# Intravital imaging of pulmonary lymphatics in inflammation and metastatic cancer

**DOI:** 10.1084/jem.20241359

**Published:** 2025-02-19

**Authors:** Simon J. Cleary, Longhui Qiu, Yurim Seo, Peter Baluk, Dan Liu, Nina K. Serwas, Catherine A. Taylor, Dongliang Zhang, Jason G. Cyster, Donald M. McDonald, Matthew F. Krummel, Mark R. Looney

**Affiliations:** 1 Department of Medicine, University of California, San Francisco (UCSF), San Francisco, CA, USA; 2 Institute of Pharmaceutical Science, King’s College London, London, UK; 3 Department of Anatomy, Cardiovascular Research Institute, and Helen Diller Family Comprehensive Cancer Center, UCSF, San Francisco, CA, USA; 4 Department of Microbiology and Immunology, Howard Hughes Medical Institute, UCSF, San Francisco, CA, USA; 5 Westlake Laboratory of Life Sciences and Biomedicine, School of Medicine, Westlake University, Hangzhou, China; 6 Department of Pathology, UCSF, San Francisco, CA, USA; 7 Bakar ImmunoX Initiative, UCSF, San Francisco, CA, USA

## Abstract

Intravital microscopy has enabled the study of immune dynamics in the pulmonary microvasculature, but many key events remain unseen because they occur in deeper lung regions. We therefore developed a technique for stabilized intravital imaging of bronchovascular cuffs and collecting lymphatics surrounding pulmonary veins in mice. Intravital imaging of pulmonary lymphatics revealed ventilation dependence of steady-state lung lymph flow and ventilation-independent lymph flow during inflammation. We imaged the rapid exodus of migratory dendritic cells through lung lymphatics following inflammation and measured effects of pharmacologic and genetic interventions targeting chemokine signaling. Intravital imaging also captured lymphatic immune surveillance of lung-metastatic cancers and lymphatic metastasis of cancer cells. To our knowledge, this is the first imaging of lymph flow and leukocyte migration through intact pulmonary lymphatics. This approach will enable studies of protective and maladaptive processes unfolding within the lungs and in other previously inaccessible locations.

## Teaser

Lymphatic vessels in the lungs play important roles in regulating fluid balance, immune responses, and cancer progression. This article reports an intravital microscopy approach for imaging these pulmonary lymphatics. This technique can also reveal cellular dynamics in other understudied tissue structures.

## Graphical Abstract

**Figure:**
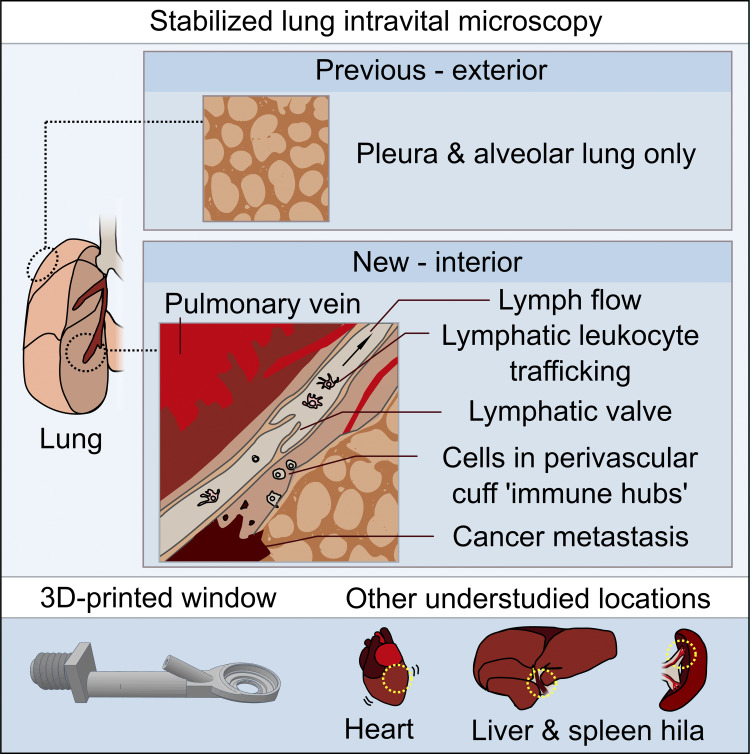

## Introduction

Stabilized intravital microscopy approaches have made it possible to directly study immune events that unfold within lung alveolar–capillary units at subcellular resolution. The use of thoracic windows to stabilize regions of lung tissue for live microscopy has enabled mechanistic insights into lymphocyte surveillance (Looney et al., 2011; Podstawka et al., 2021), neutrophil recruitment (Looney et al., 2011; Park et al., 2019; Conrad et al., 2022), neutrophil extracellular trap release (Lefrançais et al., 2018), platelet responses (Cleary et al., 2019, 2020), myeloid containment of lung-metastatic cancer cells (Headley et al., 2016), and alveolar macrophage patrolling (Neupane et al., 2020), as well as immunomodulatory and platelet-producing megakaryocytes in the lungs (Lefrançais et al., 2017; Pariser et al., 2021). Glue-based stabilization approaches have enabled imaging of immune events following lung transplantation (Kreisel et al., 2010; Li et al., 2012; Bai et al., 2024), and in lung-metastatic cancer (Entenberg et al., 2018). Transparent coverings for ex vivo lung perfusion systems have also permitted imaging across all exterior lung surfaces (Banerji et al., 2023). However, these previous approaches for live-imaging lungs have been limited to alveolar lung tissue within ∼100 µm of distal pleural surfaces, a region devoid of important structures including major airways, large blood vessels, and other structures of the lung interior.

The restriction of lung intravital microscopy to alveolar–capillary units has prevented the direct study of intact structures critical for pulmonary immune regulation. Notably, these understudied regions include bronchovascular cuff spaces that house unique leukocyte subsets and store reserves of edema fluid (Dahlgren and Molofsky, 2019). These spaces contain specialized lymphatics that transport fluid and cells out of the lungs and play vital but incompletely understood roles in lung fluid balance and immune responses in health and in various diseases (Trivedi and Reed, 2023). Intravital microscopy has proved useful for understanding the function of other specialized blood and lymphatic vessels (Dixon et al., 2006; Choe et al., 2015; Collado-Diaz et al., 2022), but research into pulmonary lymphatic function has been limited by our inability to directly image intact lymphatics in the lungs (Stump et al., 2017; Baluk and McDonald, 2022; Trivedi and Reed, 2023).

We therefore developed novel tools and approaches that have enabled direct imaging of the movement of endogenous fluid and immune cells through intact lymphatics and interstitial spaces surrounding pulmonary veins in the lungs of mice. We found that this approach can be used to answer key questions related to functions of lung lymphatic vessels in both draining fluid and leukocyte trafficking during inflammatory responses and lung-metastatic cancer. In addition, apparatus and techniques developed for studying the lungs were also found to be useful for imaging other structures previously unseen using intravital microscopy. This article reports insights into pulmonary lymphatic biology using our new technique and provides a stabilization window model that can be 3D-printed to allow other researchers to expand their studies to new tissue locations.

## Results

### An intravital microscopy approach enabling the direct study of lung lymphatic function

**Figure 1.**
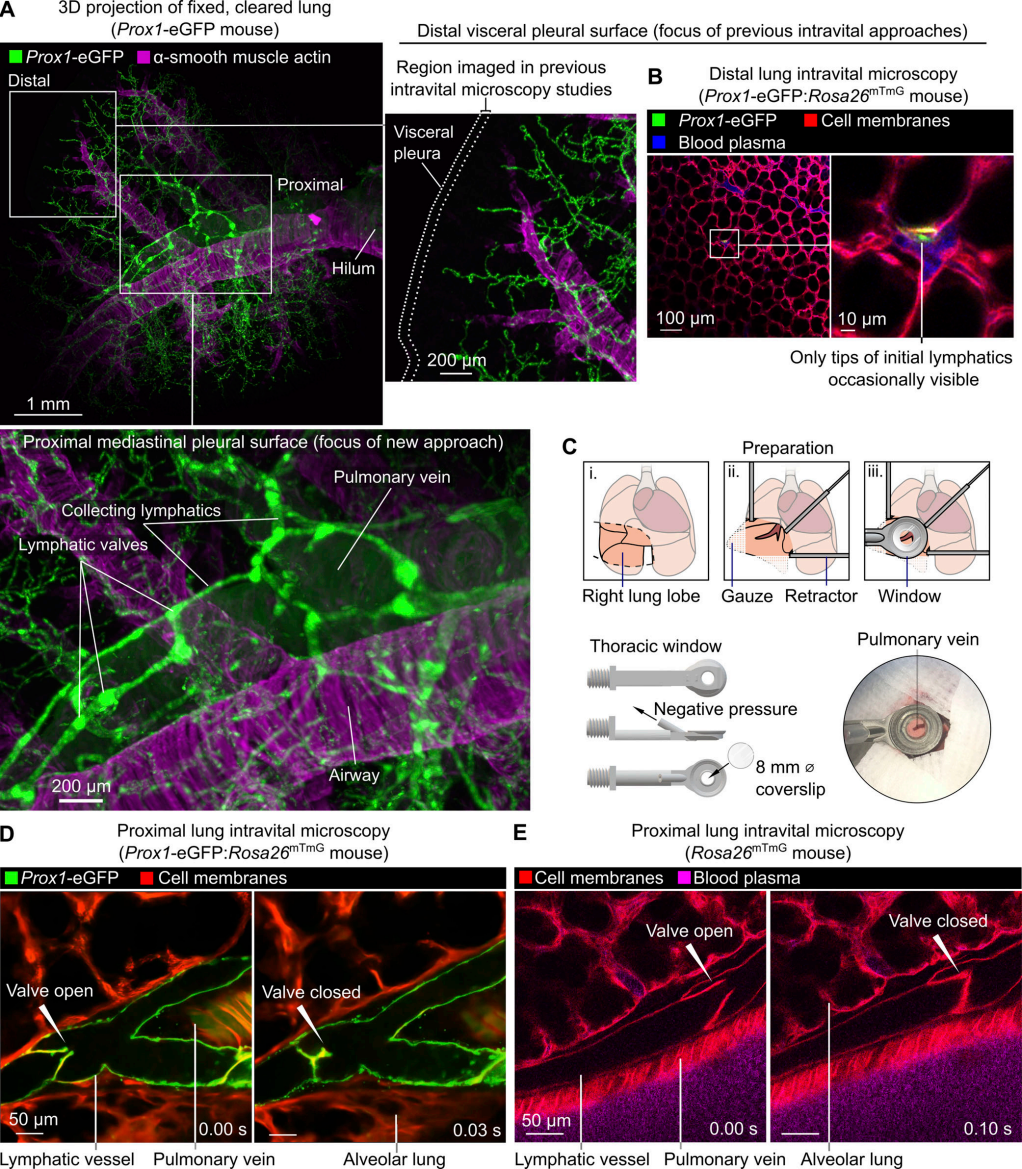
**Intravital imaging of lymphatic vessels in lungs of ventilated, anesthetized mice. (A)** 3D maximum intensity projections of the cleared lung from the *Prox1*-eGFP mouse showing paucity of lymphatics near distal pleural surfaces and prominent collecting lymphatics surrounding the pulmonary vein. **(B)** Distal lung intravital microscopy imaging showing initial lymphatic in *Prox1*-eGFP:*Rosa26*^mTmG^ mouse. **(C)** Surgical preparation, window design, and placement of window for imaging around the pulmonary vein on the mediastinal pleural surface. **(D and E)** Intravital imaging of functional collecting lymphatics in lungs of (D) *Prox1*-eGFP:*Rosa26*^mTmG^ and (E) *Rosa26*^mTmG^ mice. All images are representative of three or more independent experiments.

**Figure S1.**
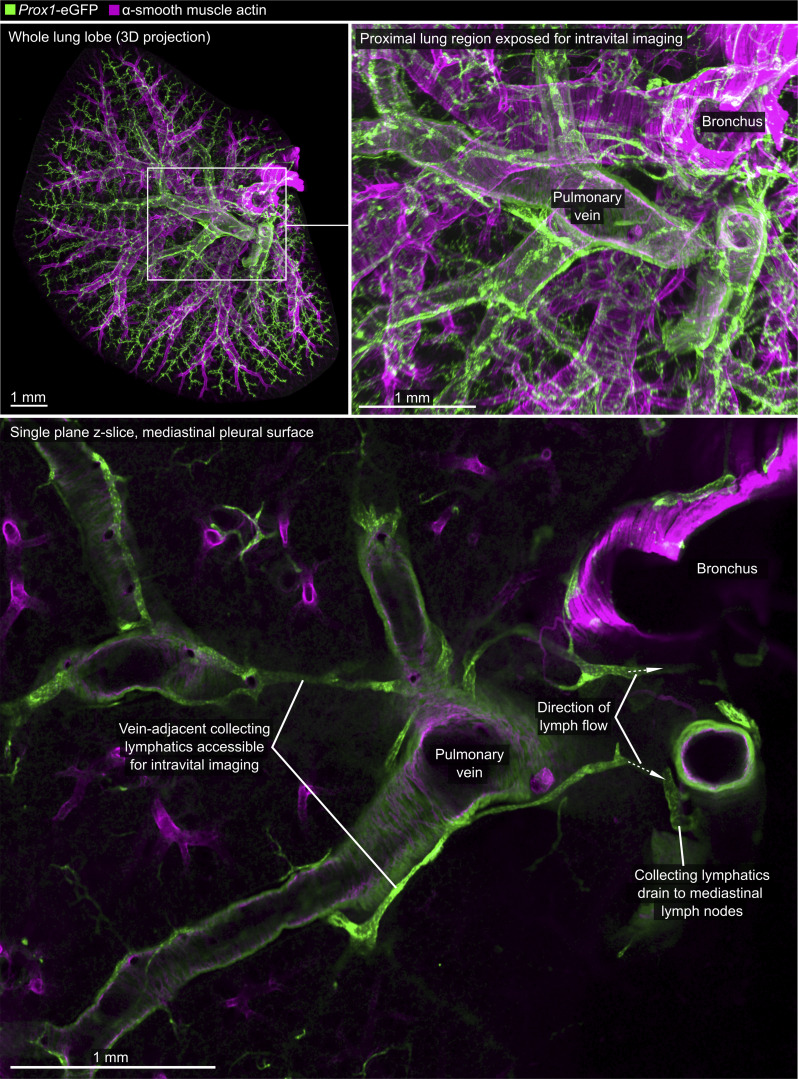
**Anatomy of pulmonary vein–associated collecting lymphatics.** The cleared lung from *Prox1*-eGFP mouse was immunostained for GFP and α-smooth muscle actin. Upper images are 3D maximum intensity projections of a whole lung lobe, with detailed expansion showing the region exposed for intravital imaging. The lower image is a single z-slice that shows lymphatics adjacent to the superficial pulmonary vein. Images are representative of cleared lungs of *n* = 4 mice.

The visceral pleural surfaces of lungs are accessible for intravital imaging but, in healthy mice, have few lymphatics. Additionally, lymphatics present in the exterior pleura are located far from the major sites of leukocyte and fluid trafficking in the lung interior (Fig. 1, A and B) (Baluk et al., 2020; Yao et al., 2014). Direct observation of the dynamics of lymphatic valves, lymph flow, and leukocyte trafficking in intact lymphatics in the lungs of living mice has therefore not been possible. Seeking an alternative location in the lungs to image lymphatics, we used cleared tissue imaging to image lymphatics across entire cleared lung lobes and observed that large collecting lymphatics follow pulmonary veins close to the proximal mediastinal surfaces of lungs before draining to mediastinal lymph nodes (Fig. 1 A and Fig. S1). In addition to lymphatics, pulmonary veins are surrounded by cardiac muscle and perivascular cuff spaces that have both been implicated in immune regulation (Folmsbee et al., 2016; Dahlgren and Molofsky, 2019), so we developed an approach to stabilize and image these structures.

To immobilize areas around superficial pulmonary veins for intravital microscopy studies, we designed a 3D-printed stabilization window with a smaller frame than previous windows used for lung imaging (Fig. 1 C and Data S1) (Looney et al., 2011; Headley et al., 2016). This window was applied with a new surgical preparation, enabling imaging of previously unseen lung structures in ventilated, anesthetized mice expressing fluorescent reporters labeling lymphatic endothelial cells (*Prox1*-eGFP) (Choi et al., 2011) and all cell membranes (*Rosa26*^mTmG^) (Muzumdar et al., 2007) (Fig. 1 D). 2D scans captured the opening and closing of pulmonary collecting lymphatic valves, pulmonary veins with pulsatile cardiac myocyte sheaths, and bronchovascular cuff spaces (Fig. 1 D and Video 1). The distinctive bicuspid valves and bronchovascular cuff location of pulmonary collecting lymphatics enabled identification of these structures without a lymphatic-restricted reporter using *Rosa26*^mTmG^ mice (Fig. 1 E and Video 1).

**Video 1.** **Ventilation dependence of pulmonary lymphatic valve function and lung** **lymph flow.**

### Lymph flow and valve dynamics in intact lung lymphatics

**Figure 2.**
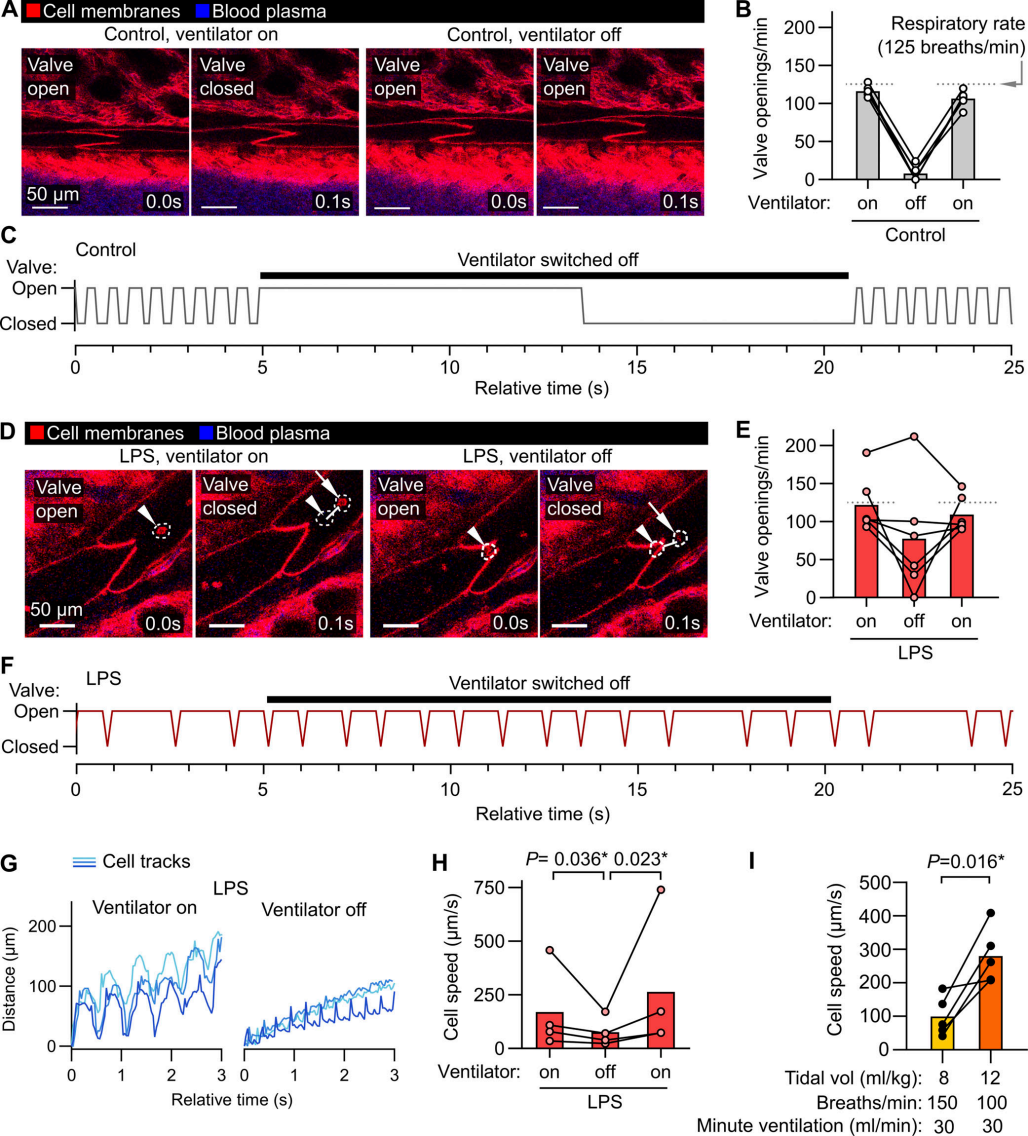
**Ventilation-dependent and ventilation-independent lymph flow through pulmonary collecting lymphatics. (A–C)** (A) Pulmonary lymphatic valves of steady-state control *Rosa26*^mTmG^ mice during ventilation and during a ventilator pause, with (B) the quantification of the effect on valve openings and (C) a representative trace of valve status over time. **(D–H)** (D) Pulmonary lymphatic valves from LPS-treated mice showing continuation of leukocyte flow and valve opening during ventilator pause, with (E) the quantification of the effect of ventilator pause on valve opening, (F) a representative valve trace, and (G) representative traces of progress of tracked leukocytes through lymphatics with (H) quantification of speeds. **(I)** Cell speeds during lower versus higher tidal volume ventilation with indicated settings. Bar graphs show means; P values are from (H) repeated-measures two-way ANOVA on log_10_-transformed data with Tukey’s multiple comparisons test; or (I) two-tailed paired *t* test. Group sizes: (B, E, and I) *n* = 5; (H) *n* = 4.

Because pulmonary collecting lymphatics typically lack smooth muscle and pericyte coverage, they are thought to be unable to generate the intrinsic peristaltic contractions that drive lymph outflow from other organs (Reed et al., 2019). These anatomical features, together with evidence that changing respiratory rate has effects on thoracic duct outflow in large animal cannulation studies (Warren and Drinker, 1942), led to the hypothesis that forces generated by ventilation primarily drive lung lymph flow. Intravital imaging of pulmonary collecting lymphatics allowed us to determine that in steady-state conditions with positive pressure ventilation, stabilized segments of pulmonary lymphatics do not display contractions but instead open and close their valves in synchrony with the respiratory rate (Fig. 2, A and B; and Video 1). Providing further evidence for a role of ventilation in driving steady-state lung lymph flow, pausing mechanical ventilation resulted in cessation of pulmonary collecting valve opening and closing (Fig. 2, A–C and Video 1). In contrast, 1 day after inducing acute lung inflammation by dosing bacterial lipopolysaccharides (LPS) into the lungs of mice, pulmonary lymphatic valves exhibited openings and closings that were asynchronous with ventilation and continued during ventilator pauses (Fig. 2, D–F and Video 1). Tracking leukocytes that had entered lung lymph flow in LPS-treated mice, we confirmed that lymph outflow from inflamed lungs continues during ventilator pauses (Fig. 2, G and H; and Video 1). Together, these findings indicate that acute inflammation leads to uncoupling of lung lymph flow from ventilation, potentially driven by increased plasma extravasation from blood vessels made leaky by inflammation, or by changes in the ability of cardiac motion or extrapulmonary lymphatic contractions to drive lung lymph flow. These findings demonstrate the importance of studying pulmonary lymphatic biology in both normal physiology and relevant disease models.

Mechanical ventilation with lower tidal volumes (6 ml/kg predicted body weight), compared with higher tidal volumes (12 ml/kg), decreases mortality in the acute respiratory distress syndrome (ARDS) (Acute Respiratory Distress Syndrome Network et al., 2000). As lung inflammation changed the ventilation dependence of lymph flow in inflamed lungs, and previous studies of the effects of tidal volume on lung lymph flow used ex vivo–perfused lungs from healthy sheep (Pearse et al., 2005), we examined the effect of ventilation with higher versus lower tidal volumes on lung lymph flow by directly imaging flow of native leukocytes in the lymph leaving LPS-inflamed lungs. We compared ventilation with higher versus lower tidal volume using settings that matched minute ventilation. Lymph flow was slow with lower tidal volume ventilation, and higher tidal volume ventilation resulted in near-immediate increases in cell speeds in lymph flow (Fig. 2 I and Video 2), highlighting coupling of pulmonary lymphatic function to lung distention. These studies demonstrate that intravital microscopy could be useful for improving our understanding of the complex interplay between lymphatic function, mechanical ventilation, and lung fluid regulation in the setting of acute lung injury.

**Video 2.** Effect of changing ventilator tidal volume on lymph flow within pulmonary lymphatics following LPS-induced acute lung inflammation.

### Leukocyte dynamics and diversity in lymphatics during lung inflammation

**Figure 3.**
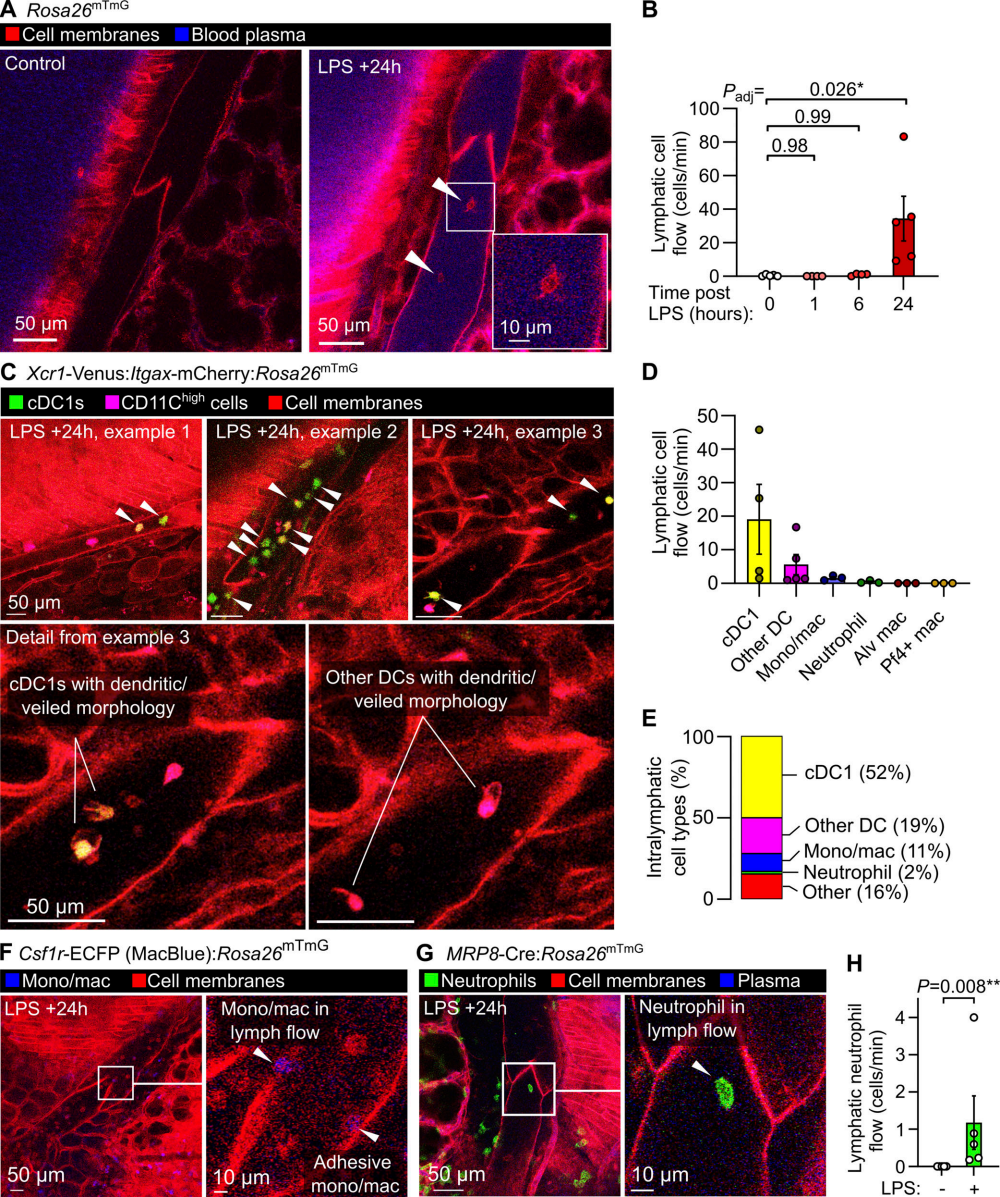
**Dynamics and diversity of leukocyte trafficking within intact pulmonary lymphatics. (A)** Pulmonary lymphatic vessels from a steady-state control and LPS-treated *Rosa26*^mTmG^ mice at 24 h after onset of LPS-induced lung inflammation with arrowheads pointing to intralymphatic leukocytes. **(B)** Quantification of lymphatic flow of leukocytes. **(C–H)** Pulmonary lymphatics in *Xcr1*-Venus:*Itgax*-mCherry:*Rosa26*^mTmG^ mice at 24 h after LPS treatment with arrowheads pointing to *Xcr1*-Venus+ cDC1s, with cell types in lymphatics quantified in D and E using the mouse lines shown in this figure and in Fig. S2. Pulmonary lymphatic vessels from (F) *Csf1r*-ECFP:*Rosa26*^mTmG^ monocyte/macrophage reporter mouse or (G) *MRP8*-Cre:*Rosa26*^mTmG^ neutrophil reporter mouse 24 h after LPS treatment with (H) quantification of lymphatic flow of neutrophils. Graphs show means ± SEM. P values are from (B) the Kruskal–Wallis test with Dunn’s multiple comparisons to the 0-h (naïve) group; or (H) the Mann–Whitney test. Group sizes: (B) *n* = 4 (+1- and +6-h groups), *n* = 5 (0- and +24-h groups); (D and E) *n* = 3–5; (H) *n* = 5. Alv macs, alveolar macrophages.

**Figure S2.**
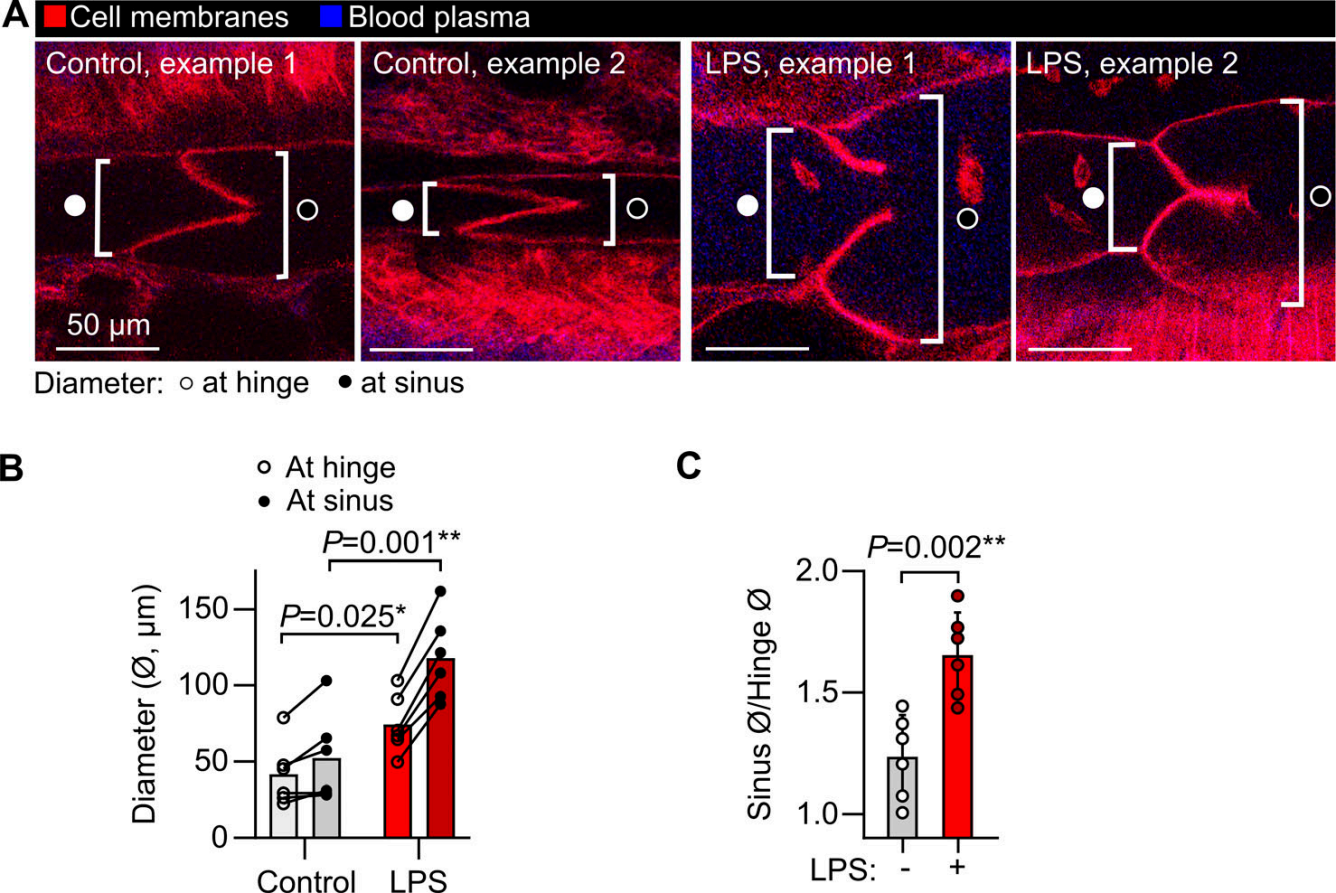
**Measurement of pulmonary lymphatic distension in LPS-induced acute lung inflammation. (A)** Representative images of pulmonary lymphatic valves from steady-state controls and LPS-treated *Rosa26*^mTmG^ mice showing an approach for measuring lymphatic diameter. **(B)** Lymphatic vessel diameters at valve hinges and at sinuses immediately downstream of valves. **(C)** Sinus diameters divided by hinge diameters showing the relative distension of sinuses. Graphs show means ± SEM. P values are from (B) repeated-measures, two-way ANOVA with the Holm–Šídák test for the effect of LPS within vessel region groups; or (C) unpaired, two-tailed *t* test. Group sizes: *n* = 6.

Previous intravital studies of lymphatics draining the skin and mesentery have revealed a stepwise process involving migration of leukocytes into lymphatic vessels (Pflicke and Sixt, 2009), followed by leukocyte crawling on the luminal lymphatic endothelial surface (Collado-Diaz et al., 2022), then leukocyte detachment for entry into lymph flow (Dixon et al., 2006). These events are important for adaptive immunity and immune tolerance but have not been characterized using live imaging in intact lung lymphatics. Additionally, determining the cellular contents of lung lymph has been challenging using currently available approaches, particularly in small animals (Ying et al., 1994; Baluk et al., 2020; Stolley et al., 2020; Tang et al., 2022). Using our intravital imaging approach, we found that 24 h after onset of LPS-induced lung inflammation, many leukocytes in collecting lymphatics had entered lymph flow, moving at speeds of 25–500 µm/s (Fig. 2 I; Fig. 3, A and B; Video 1; and Video 2). Not all intralymphatic leukocytes flowed freely in the lymph. Some were observed rolling on and becoming adhesive to the lymphatic endothelium (Video 3), indicating that, mirroring the leukocyte adhesion cascade in blood vessels, a similar set of processes may also enable immune surveillance within pulmonary lymphatics. Lung lymphatics became distended in response to LPS (Fig. S2), and lymphatic transport of the extravasated plasma protein could be imaged using intravenously (i.v.) injected Evans blue dye (Fig. 2 A and Video 3).

**Video 3.** Leukocyte rolling and adhesion within pulmonary lymphatics.

A large fraction of the cells entering lymph flow were dendritic cells with visible dendritic or veiled morphology, confirmed by imaging mice expressing the *Xcr1*-Venus reporter (labeling type 1 conventional dendritic cells) and *Itgax*-mCherry (labeling the majority of dendritic cells) (Fig. 3, C–E and Video 4) (Cabeza-Cabrerizo et al., 2021). Monocyte/macrophage cells with the high expression of the Csf1r-eCFP reporter were also imaged within lymphatics (Fig. 3, E and F; and Video 4). Neutrophils have been observed in lymphatic vessels (Rigby et al., 2015; Lok et al., 2019), and using *MRP8*-Cre:*Rosa26*^mTmG^ neutrophil reporter mice, we quantified neutrophil trafficking in pulmonary lymphatics after LPS treatment (Fig. 3, G and H; and Video 4).

**Video 4.** Dendritic cells, monocytes/macrophages, and neutrophils in pulmonary lymphatics after LPS-induced acute lung inflammation.

**Figure S3.**
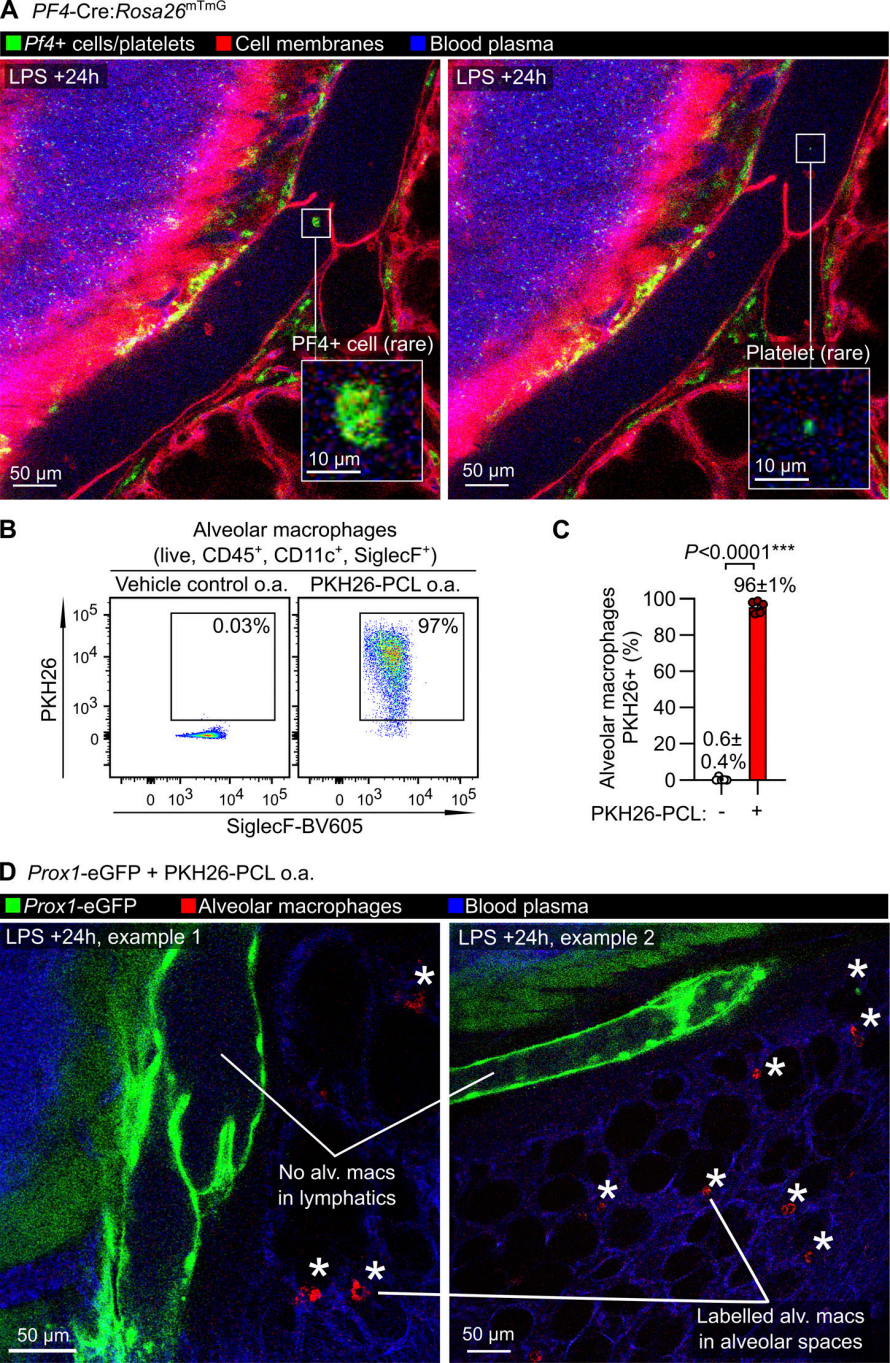
**Imaging of pulmonary lymphatics in LPS-treated *PF4*-Cre:*Rosa26*** ^**mTmG**^ **mice and *Prox1*-eGFP mice given PKH26-PCL to label alveolar macrophages. (A)** Intravital images of an LPS-treated *PF4*-Cre:*Rosa26*^mTmG^ mouse showing platelets in blood vessels and recombined cells in bronchovascular cuff spaces but only very rare recombined cells and possible platelets in the lymph. **(B and C)** C57BL/6 mice were given PKH26-PCL or vehicle control o.a., and 5 days later, BAL cells were isolated for measurement of efficiency of labeling of alveolar macrophages with PKH26, quantified in C. **(D)***Prox1*-eGFP mice were given PKH26-PCL o.a. to label alveolar macrophages, then 5 days later were given LPS via the o.a. route. Intravital imaging at 24 h after LPS treatment showed labeling of alveolar macrophages (alv. macs, asterisks) in alveoli but not in lymphatic vessels. The P value in C is from an unpaired, two-tailed *t* test, group sizes: (A) *n* = 3; (B–D) *n* = 5.

Using the *PF4*-Cre:*Rosa26*^mTmG^ line, with labeling of megakaryocytes and platelets in the lungs, we observed only rare entry of platelet-sized particles into lung lymph flow following LPS treatment (Fig. S3 A and Video 5). As the *Itgax*-mCherry reporter also labels alveolar macrophages, and alveolar macrophages have been reported as trafficking to lung-draining lymph nodes but not imaged directly (Kirby et al., 2009), we labeled alveolar macrophages with PKH26 dye aggregates 5 days prior to imaging (Neupane et al., 2020), but did not observe alveolar macrophages entering lymphatics during the inflammatory response to LPS (Fig. S3, B–D and Video 5).

**Video 5.** Absence of alveolar macrophages and PF4+ cells from pulmonary lymphatics after LPS-induced acute lung inflammation.

### Effects of interventions altering lymphatic trafficking of leukocytes

**Figure 4.**
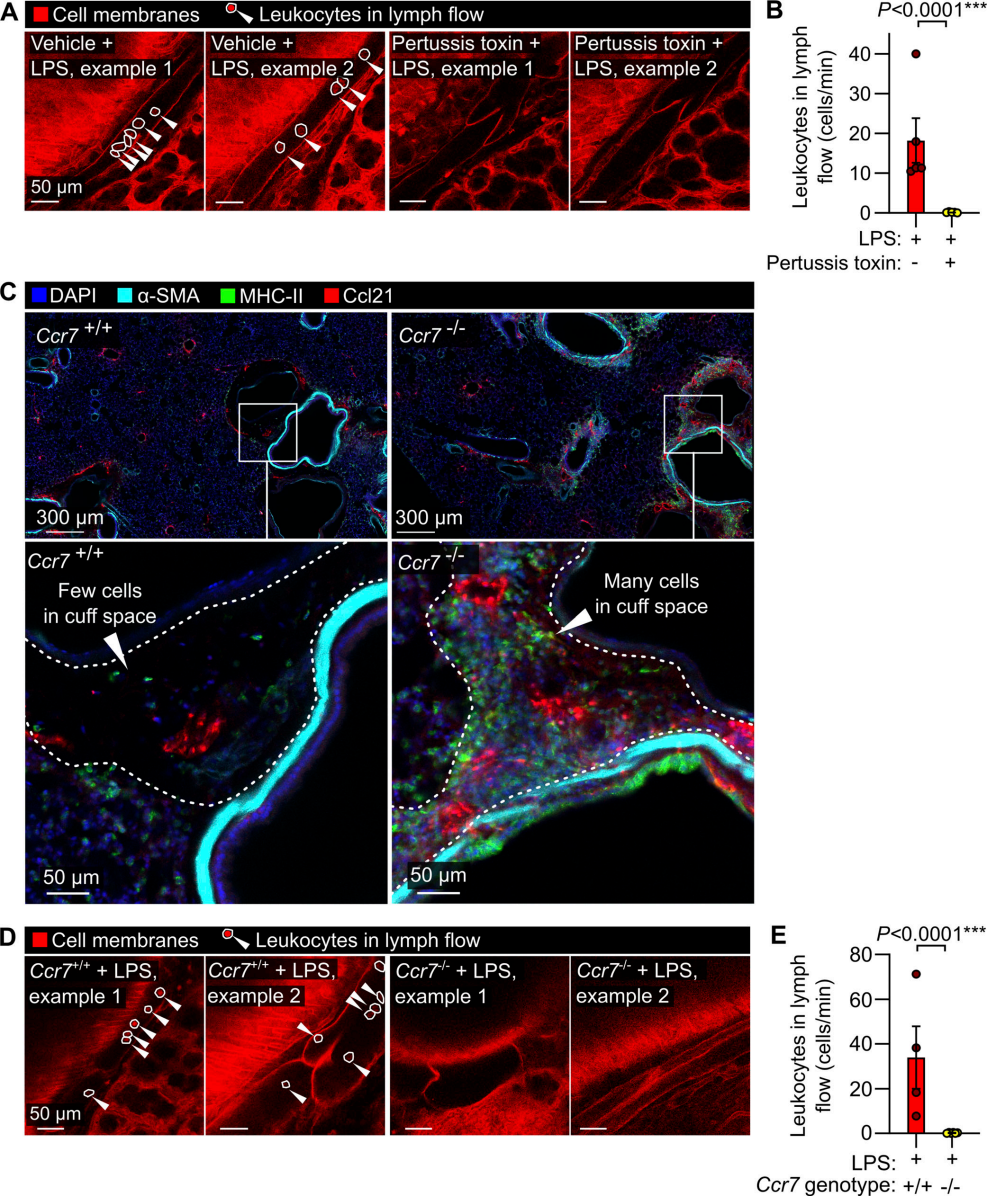
**Leukocyte trafficking through lung lymphatics requires Gαi signaling and Ccr7 expression. (A and B)** Leukocyte flow through pulmonary lymphatics from *Rosa26*^mTmG^ mice treated with either vehicle control or pertussis toxin before challenge with LPS with the quantification of the effect of pertussis toxin in B. **(C)** Immunofluorescence images of lung sections from *Ccr7*^+/+^ wild-type mice and *Ccr7*^−/−^ knockouts showing accumulation of MHC-II+ cells in bronchovascular cuffs. **(D and E)** Pulmonary lymphatic leukocyte flow in LPS-treated *Ccr7*^+/+^:*Rosa26*^mTmG^ mice and the absence of intralymphatic leukocytes in *Ccr7*^−/−^:*Rosa26*^mTmG^ mice, with (E) the quantification of the effect of Ccr7 knockout. White arrowheads in A and D point to intralymphatic leukocytes (also circled in white). Graphs show means ± SEM. P values are from unpaired, two-tailed *t* tests on log_10_-transformed datasets. Group sizes: (B) *n* = 5; (C and E) *n* = 4.

Mechanistically, G_αi_ protein–coupled receptors including Ccr7 have been implicated in lymphatic trafficking of leukocytes in mice (Saeki et al., 1999; Hammad et al., 2003; Czeloth et al., 2005). We inhibited signaling through G_αi_ subunits in mice using pertussis toxin, which eliminated lymphatic trafficking of immune cells in response to LPS inhalation (Fig. 4, A and B; and Video 6). *Ccr7* knockout leads to reduced leukocyte trafficking to lymph nodes, development of leukocyte aggregates in the lungs, and defects in immune tolerance (Fleige et al., 2018). We confirmed that the bronchovascular cuff spaces in lungs *of Ccr7*^−/−^ mice become filled with leukocytes (Fig. 4 C), and found that knockout of *Ccr7* greatly reduced leukocyte trafficking via pulmonary lymphatics 1 day after LPS treatment (Fig. 4, D and E; and Video 7).

**Video 6.** Effect of pertussis toxin on leukocyte flow within pulmonary lymphatics following LPS-induced acute lung inflammation.

**Video 7.** Effect of knockout of *Ccr7* on leukocyte flow within pulmonary lymphatics following LPS-induced acute lung inflammation.

**Figure 5.**
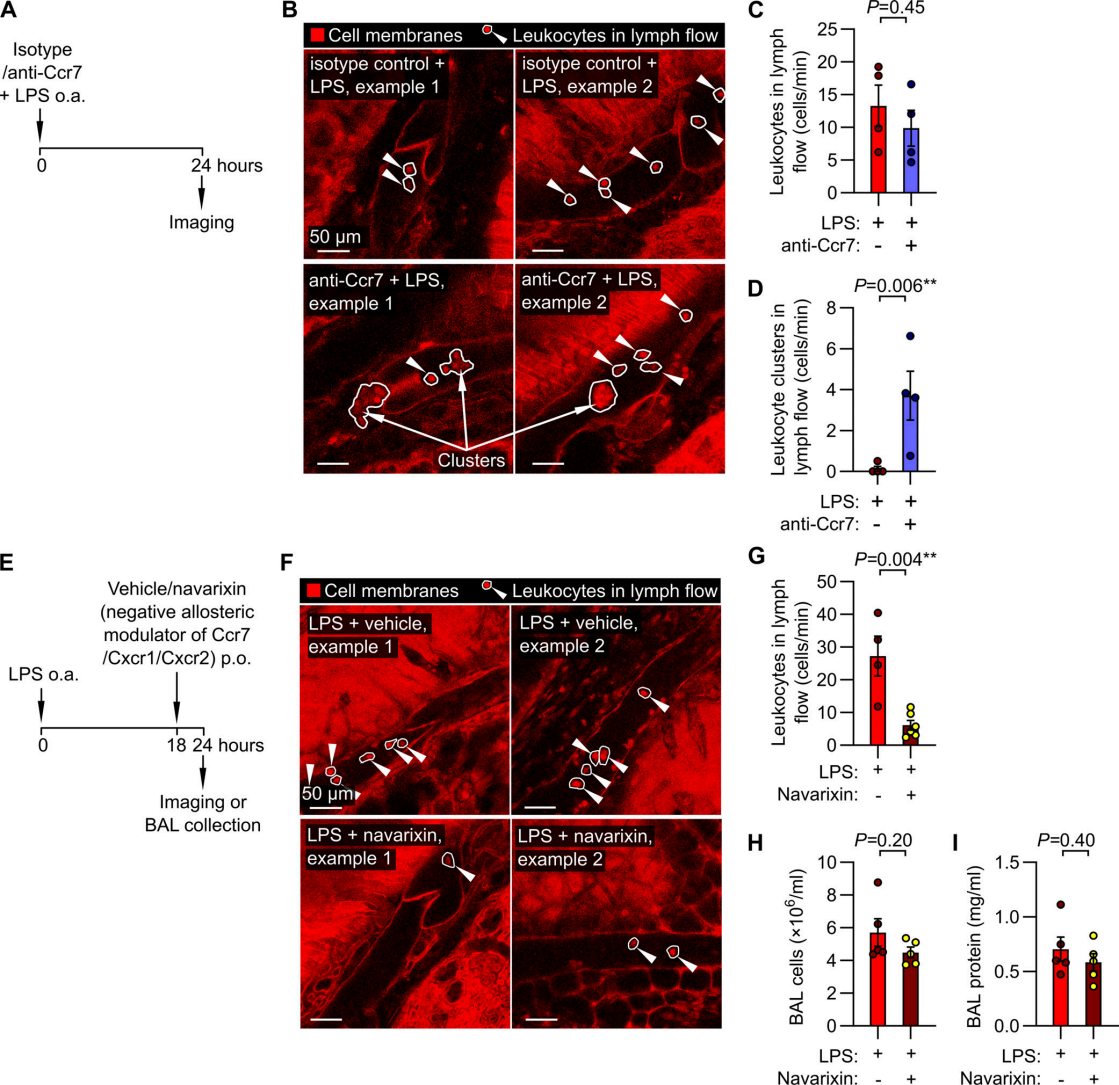
**Effects of Ccr7 inhibition on leukocyte trafficking via pulmonary lymphatics. (A)** *Rosa26* ^mTmG^ mice were given either isotype-matched control antibody or anti-Ccr7 by o.a. together with LPS. **(B–D)** Leukocytes continued to flow through lung lymphatics in anti-Ccr7–treated mice, quantified in C, but these leukocytes formed intralymphatic multicell clusters, quantified in D. **(E)** Separate mice were administered LPS, and 18 h later were randomized to receive navarixin (5 mg/kg by oral gavage [p.o.]) or vehicle control. **(F–I)** At 24 h after LPS administration, treatment with navarixin reduced leukocyte flow through pulmonary lymphatics, quantified in G. This navarixin dosing strategy did not alter the extent of lung inflammation readouts of BAL fluid: (H) cell content or (I) protein concentration. White arrowheads in B and F point to intralymphatic leukocytes (also circled in white), and white arrows in B point to multicell intralymphatic leukocyte clusters. P values are from unpaired, two-tailed *t* tests on log_10_-transformed datasets. Group sizes: (C and D) *n* = 4; (G) *n* = 4-6; (H and I) *n* = 5.

Antibodies targeting human CCR7 are under clinical investigation, and antibodies targeting mouse Ccr7 have been used as research tools (Cuesta-Mateos et al., 2021). To understand how these agents might be altering pulmonary lymphatic function, we tested the effect of a functionally blocking Ccr7 antibody on lymphatic leukocyte trafficking. Delivery of this Ccr7 antibody directly into the lungs together with LPS did not prevent the entry of leukocytes into pulmonary collecting lymphatics but instead caused the appearance of large clusters of leukocytes that still achieved entry into lymph flow (Fig. 5, A–D and Video 8).

**Video 8.** Effect of *Ccr7* blocking antibody treatment on leukocyte flow within pulmonary lymphatics following LPS-induced acute lung inflammation.

Inhaled delivery of Ccr7 antibody may have incompletely blocked Ccr7 on interstitial leukocytes but allowed sufficient quantities of the Ccr7 antibody to enter lymphatics to cause Fc receptor–mediated agglutination of intralymphatic leukocytes. We therefore tested an alternative Ccr7 inhibition approach using navarixin, a small molecule negative allosteric modulator of Ccr7, Cxcr1, and Cxcr2 that has been studied in clinical trials for airway disease and cancer indications (Jaeger et al., 2019; Rennard et al., 2015; Armstrong et al., 2024). Treatment with navarixin 18 h after LPS administration decreased the number of leukocytes flowing in lung lymphatics (Fig. 5, E–G and Video 9). This effect was unlikely to be due to a reduction in lung inflammation, as navarixin treatment after onset of acute lung inflammation did not alter leukocyte recruitment or protein extravasation into lung airspaces (Fig. 5, H and I). These findings are consistent with a role of Ccr7 in lymphatic emigration of lung-resident leukocytes during acute lung inflammation. Together, these results demonstrate that our intravital microscopy approach can be used to determine mechanisms of leukocyte trafficking through pulmonary lymphatics.

**Video 9.** Effect of navarixin treatment on leukocyte flow within pulmonary lymphatics following LPS-induced acute lung inflammation.

### Lymphatic immune surveillance of metastatic tumors

**Figure 6.**
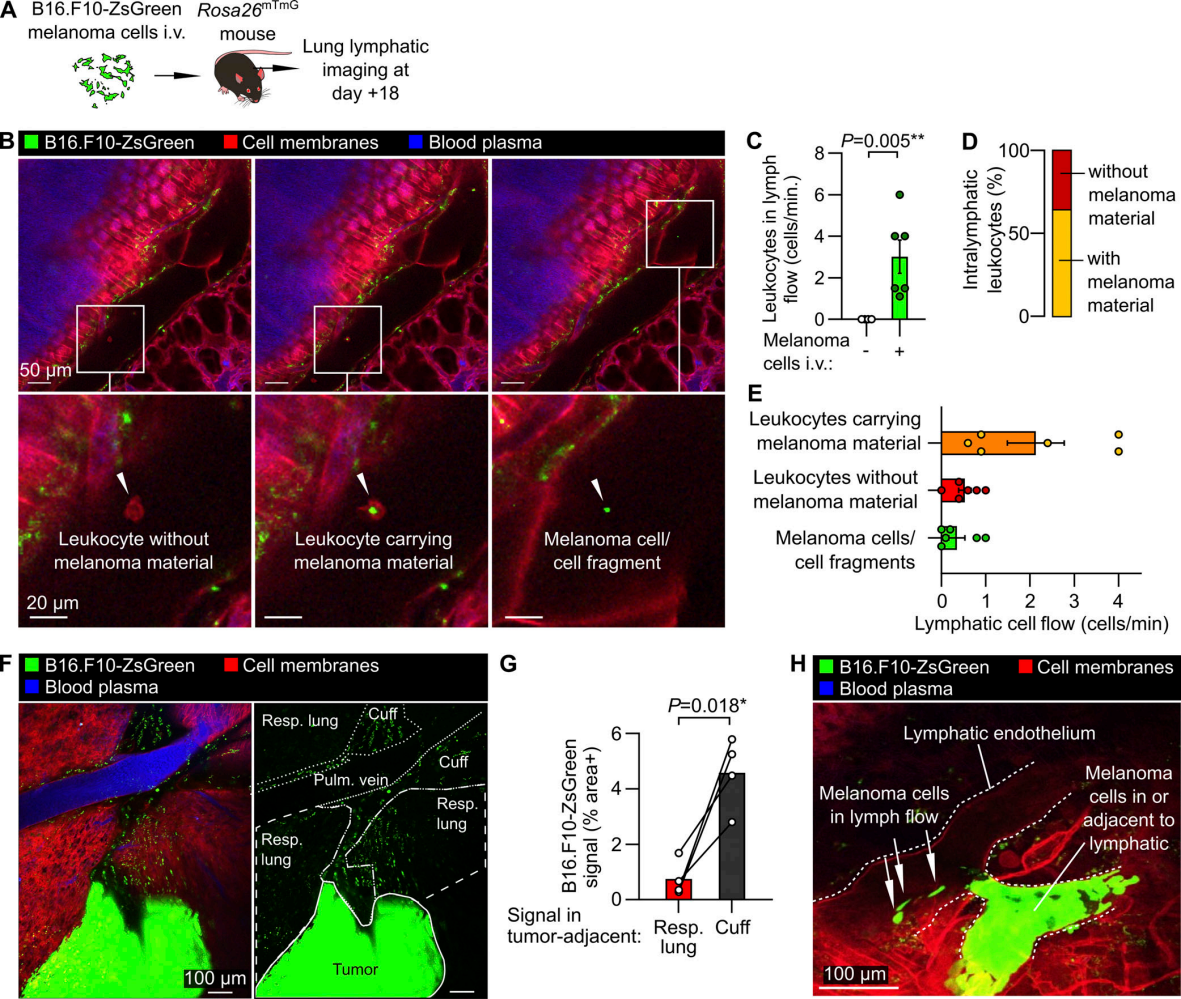
**Tumor–immune interactions and cancer cell material within pulmonary lymphatics. (A)** Schematic diagram summarizing the B16.F10-ZsGreen melanoma mouse model. **(B–E)** Images of intralymphatic leukocytes without melanoma material, leukocytes carrying melanoma material, and melanoma cell/cell fragments in lymph flow, with (C) the quantification of total leukocyte flow and (D and E) proportions of cells observed in lymphatics. **(F and G)** (F) Overview showing metastatic tumor in lung with (G) the quantification of enrichment of bronchovascular cuff space (“cuff”) relative to respiratory (“resp.”) lung. **(H)** Image showing melanoma cells in a lymphatic vessel adjacent to an accumulation of melanoma material either in or adjacent to a lymphatic. Graphs show means ± SEM. P values are from (C) a Mann–Whitney U-test; and (G) a two-tailed paired *t* test. Group sizes: (B and D) *n* = 6, (C) *n* = 4–6, (F) *n* = 4.

**Figure S4.**
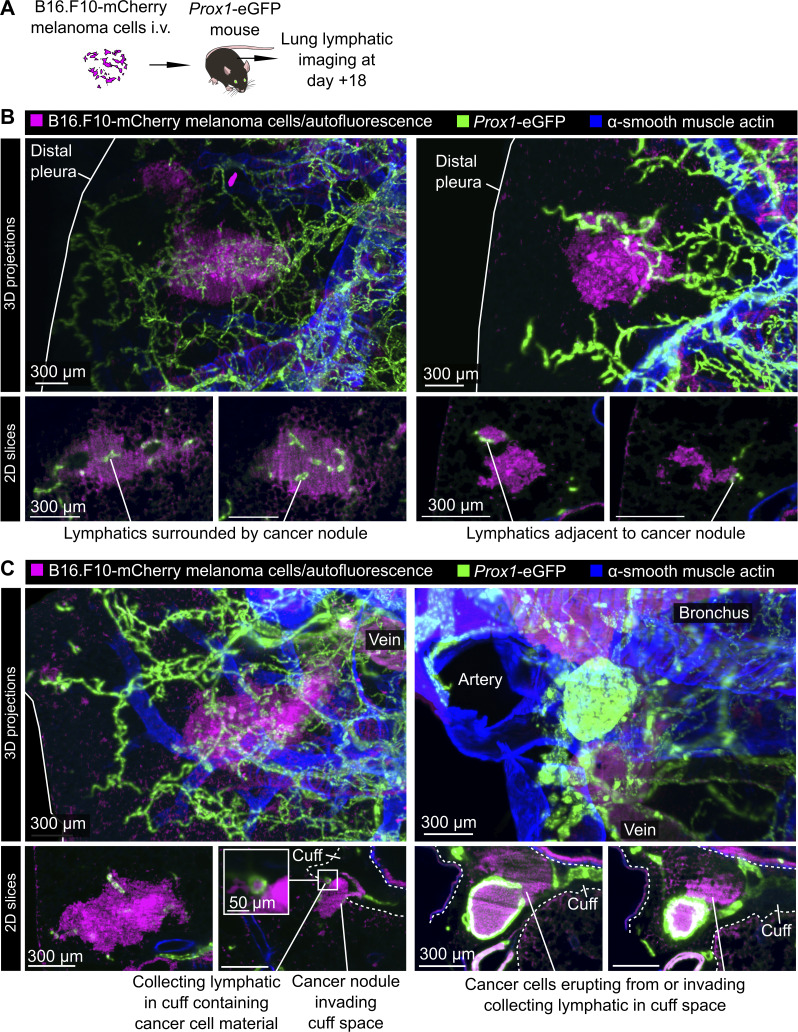
**3D imaging of pulmonary lymphatics in metastatic melanoma. (A)** Lungs were collected from *Prox1*-eGFP mice 18 days after i.v. B16.F10-mCherry melanoma cell injections. **(B)** Lungs were fixed, cleared, and immunostained for visualization of melanoma metastases, the pulmonary lymphatic vasculature, and smooth muscle surrounding airways and arteries in 3D using light-sheet microscopy. Images show 3D maximum intensity projections of subpleural metastases above single z-slices from the same nodules showing their association with initial lymphatic vessels. **(C)** 3D maximum intensity projections and matched single z-slice images showing examples of cancer cell material inside bronchovascular cuff and collecting lymphatics. Images are representative of three independent experiments.

Lymphatic-dependent immune responses, lymphatic metastasis, and lymphangiogenesis have been linked to altered cancer outcomes (Shields et al., 2010; Ma et al., 2018; Ubellacker et al., 2020; Steele et al., 2023). We therefore developed protocols for imaging immune surveillance and dissemination of metastatic cancer cells via pulmonary lymphatics. We modeled lung metastasis by i.v. injecting mice with B16.F10 melanoma cells engineered to express fluorescent proteins to allow detection of cancer cells, their subcellular fragments, and cancer cell material taken up by leukocytes (Headley et al., 2016; Ruhland et al., 2020; Yi et al., 2022). Intravital imaging of collecting lymphatics of *Rosa26*^mTmG^ mice 18 days after seeding of lungs with B16.F10-ZsGreen cells enabled quantification of increased flow of leukocytes in lymphatics in mice with lung-metastatic cancer (Fig. 6, A–C and Video 10). The majority (approximately two thirds) of intralymphatic leukocytes contained material from cancer cells (Fig. 6 D), indicating active lymphatic immune surveillance despite the failure of immune responses to clear tumors in this model without immunotherapy interventions (Ya et al., 2015). Intravital imaging also captured lymphatic metastasis of cancer cells (Fig. 6, B and E; and Video 10), and revealed enrichment of bronchovascular cuff spaces with cancer cell material (Fig. 6, F and G), and, in one mouse, signal from cancer cells either in or adjacent to a collecting lymphatic containing cancer cell material (Fig. 6 H). To better understand the relation of pulmonary lymphatics to lung metastases in 3D, we also imaged fixed, cleared lungs of *Prox1*-eGFP mice 18 days after seeding with B16.F10-mCherry cancer cells. Lung metastases were mostly in subpleural locations, often surrounding or adjacent to initial lymphatics (Fig. S4, A and B). Cancer cells were also observed inside veins and collecting lymphatics in proximal bronchovascular cuff spaces (Fig S4 C). Imaging studies of immune surveillance and metastatic dissemination within pulmonary lymphatic and interstitial niches could therefore be used to improve our mechanistic understanding of cancer pathogenesis and treatment.

**Video 10.** Pulmonary lymphatic trafficking of leukocytes, cancer cell material, and cancer cells following lung metastasis of B16.F10 melanoma cells.

### Imaging lymphatics draining other organs

**Figure S5.**
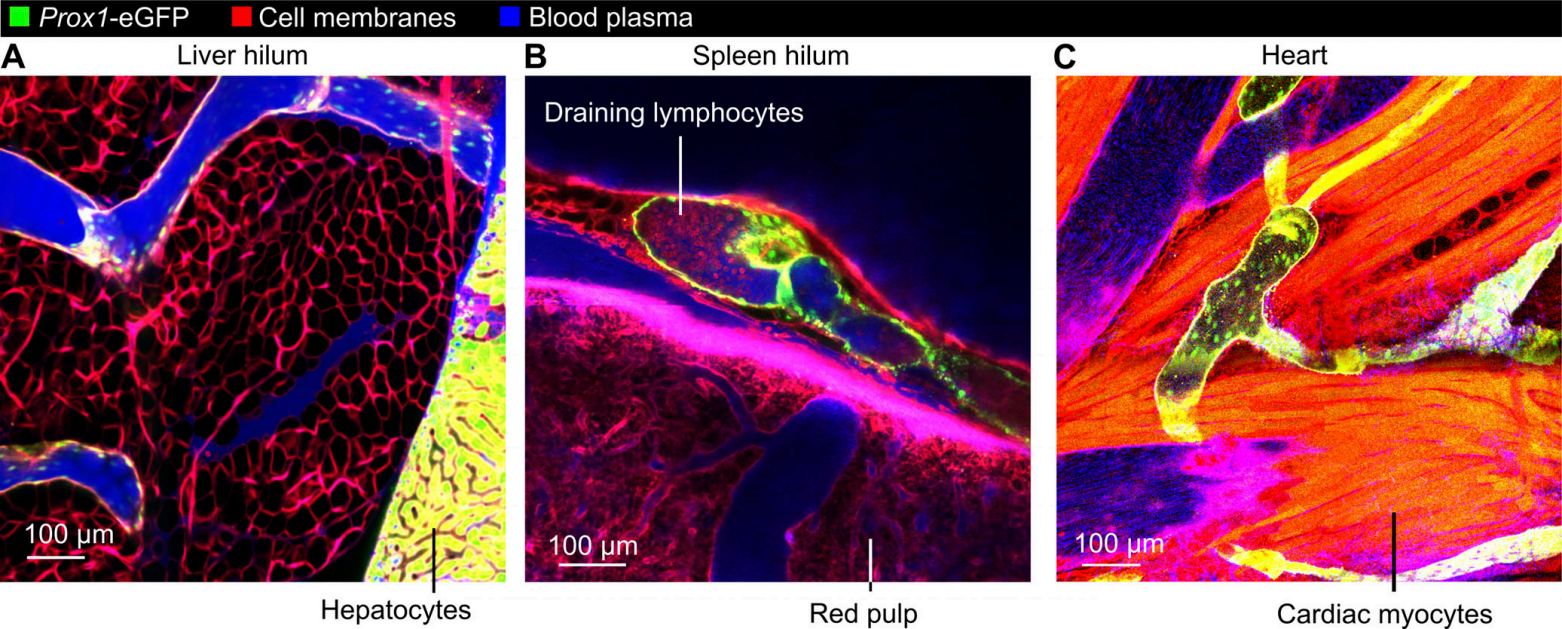
**Stabilized imaging of lymphatic vessels draining the liver, spleen, and heart. (A–C)** *Prox1*-eGFP:*Rosa26*^mTmG^ mice were given Evans blue i.v. prior to stabilized intravital imaging of (A) the hilum of the liver, (B) the hilum of the spleen, and (C) the ventricular wall of the heart. Note free movement of Evans blue–labeled plasma proteins into liver and spleen draining lymphatics, likely due to the fenestrated endothelium lining blood vessels in these organs, as well as many leukocytes with lymphocyte morphology draining from the spleen, a secondary lymphoid organ. Images are representative of three independent experiments.

Lastly, we tested whether our stabilization window could also be useful for imaging other tissues that are challenging to access and stabilize. With a similar approach, we imaged lymphatics in the hepatic hilum near the point of entry of the portal vein (Fig. S5 A and Video 11). We also imaged lymphatics draining the spleen, where leukocytes with lymphocyte morphology were abundant under normal conditions (Fig. S5 B and Video 11). In addition, the window developed in this study also enabled imaging of lymphatics within the beating heart (Fig. S5 C and Video 11). The stabilization approach reported in this study can therefore be used for intravital microscopy experiments in a diverse range of other understudied tissues and organs.

**Video 11.** Intravital imaging of lymphatics draining the liver, spleen, and heart.

## Discussion

To our knowledge, the method reported in this study has enabled the first direct visualization of cellular dynamics within intact pulmonary lymphatics and bronchovascular cuff spaces. This new intravital microscopy approach solves several problems that have limited previous studies of pulmonary lymphatic function. Lung intravital microscopy has previously only been applied to the distal alveolar microvasculature, whereas this new method enables imaging of collecting lymphatics, bronchovascular cuff spaces, and pulmonary veins, each of which has specialized and disease-relevant features that warrant direct study (Dahlgren and Molofsky, 2019; Trivedi and Reed, 2023; Baluk and McDonald, 2018, 2022). Approaches that have been established for studying pulmonary lymphatic function have involved excision of lungs, lymphatic vessels, or lymph nodes, the cannulation of extrapulmonary lymphatics, or microinjections into lung interstitial spaces (Reed et al., 2019; Dahlgren and Molofsky, 2019; Folmsbee and Gottardi, 2017). In our method, but not in these previous approaches, lymphatic function can be studied with continual ventilation, perfusion, and innervation, as well as intact flow through lymph nodes and thoracic duct outflow into the bloodstream. The use of genetically encoded fluorophores for monitoring cell trafficking and lymph flow also avoids potential artifacts from effects of tracer injections into delicate lung air or interstitial spaces, or ex vivo manipulation and adoptive transfer of cells.

Our findings also highlight how direct intravital imaging can reveal previously unappreciated effects of interventions. Specifically, leukocyte-agglutinating effects of antibodies targeting Ccr7 have not previously been observed in studies using this antibody in vivo (Peske et al., 2015; McNamee et al., 2015; Hellmann et al., 2016; Pei et al., 2019; Liu et al., 2023). Additionally, effects of navarixin on leukocyte trafficking through lymphatics were not anticipated when navarixin was taken into clinical trials for asthma and chronic obstructive pulmonary disease (Holz et al., 2010; Todd et al., 2016; Rennard et al., 2015), or when navarixin was initially studied in cancer models (Li et al., 2019).

The requirement of positive pressure ventilation is a limitation of our approach, although this feature is of relevance to the millions of people with conditions such as ARDS worldwide, annually, who receive supportive care from mechanical ventilation (Wunsch et al., 2010). Intravital microscopy studies may be useful for improving our understanding of how pulmonary lymphatic contributions to lung fluid balance are affected by different ventilation approaches (e.g., sighs, recruitment maneuvers, and high-frequency oscillatory ventilation strategies) and drugs used to treat pulmonary edema. Our stabilization approach requires the application of gentle suction, but involves the use of pressures that do not cause inflammation (Cleary et al., 2020; Conrad et al., 2022). Our imaging is currently limited to 2D scans to achieve sufficient frame rates to track leukocytes in lymphatic flow, although 3D imaging of these fast-moving cells may be possible with improved microscopy technology. Motion artifacts from separate sources (the pulse of the cardiomyocyte sheath surrounding the pulmonary vein, and the respiratory cycle) remain a challenge, as current tools for postacquisition image stabilization cannot correct for movement of compliant lung tissue in multiple directions within the same field of view. We used mice in this study to allow the use of genetic modifications, but similar approaches will likely be useful in other model organisms, particularly as transgenic reporters are increasingly available in other animals, e.g., the *Prox1*-eGFP rat line (Jung et al., 2017).

Intravital microscopy studies using this method will be useful for investigating emerging concepts in lymphatic biology, including intralymphatic coagulation (Summers et al., 2022; MacDonald et al., 2022), lymphatic junctional plasticity (Baluk and McDonald, 2022; Churchill et al., 2022), pulmonary lymphangiogenesis (Baluk et al., 2020; Szőke et al., 2021), differential molecular regulation of leukocyte entry into initial versus collecting lymphatics (Arasa et al., 2021), and the incompletely understood role of lymphatics in major lung diseases including COVID-19, asthma, pulmonary fibrosis, and tuberculosis (Trivedi and Reed, 2023). Beyond the lymphatic system, the versatile stabilization window that we developed for this study will also be useful for revealing unseen biology in a range of other tissues that are either difficult to access or challenging to image due to intrinsic motility.

## Materials and methods

### Thoracic window production and assembly

Thoracic windows were 3D-printed in high-detail stainless steel using metal binder jetting (3D model provided as Data S1). After polishing of the steel frame, an 8-mm #1 round coverslip (Cat# 64-0701; Thomas Scientific) was inserted into the immersion liquid holder and sealed by using a needle to apply epoxy resin onto the outer edges of the coverslip and supporting steel surface. Following overnight drying, sealing of the coverslip onto the steel frame was confirmed by checking for retention of water added to the immersion liquid holder during aspiration through the suction port. Thoracic windows were cleaned with Terg-a-zyme (Cat# Z273287; Sigma-Aldrich), sprayed with 70% ethanol, and rinsed with sterile deionized water.

### Animal studies

Animal studies were conducted with approval from the University of California, San Francisco (UCSF) institutional animal care and use committee. Male and female mice were used at ages 6–16 wk, and all mice were bred and maintained in the specific pathogen–free facility at UCSF. *Prox1*-eGFP mice (Choi et al., 2011) were from Donald M. McDonald (UCSF, San Francisco, CA, USA). *Rosa26*^mTmG^ mice were from Jax (Cat# 007576) (Muzumdar et al., 2007). *Xcr1*-Venus mice (Yamazaki et al., 2013), *Itgax* (CD11c)-mCherry mice (Khanna et al., 2010), and MacBlue mice (Sauter et al., 2014) were from Matthew F. Krummel (UCSF, San Francisco, CA, USA). *MRP8*-Cre (Passegué et al., 2004) and *PF4*-Cre (Tiedt et al., 2007) mice were from Jax (Cat# 021614 and Cat# 008535, respectively). All mice were backcrossed >10 generations onto the C57BL/6 background, and comparisons were made with littermate controls. As previously described, Evans blue dye (3 mg/kg, 0.75 mg/ml in 100 μl PBS) was injected i.v. immediately prior to imaging to label blood plasma proteins (Cleary et al., 2020). To induce acute lung inflammation, we administered mice a single dose of LPS (O55:B5, Cat# L2880; Sigma-Aldrich) at 4 mg/kg in PBS by oropharyngeal aspiration (o.a.) (Seo et al., 2023; Conrad et al., 2022). PKH26-phagocytic cell linker was given by o.a. as a 0.5 µM solution at 75 μl per mouse 5 days before imaging to label alveolar macrophages (Neupane et al., 2020). Pertussis toxin (Cat# P2980-50UG; Sigma-Aldrich) was given i.v. immediately after LPS dosing at 1 µg per mouse in 100 μl PBS. Functional grade anti-Ccr7 clone 4B12 was purchased from Invitrogen (Cat# 50-144-95), compared to treatment with a nonreactive isotype-matched control clone 2A3 (Cat# BE0089; Bio X Cell), given together with LPS by o.a. at 50 µg per mouse in a total volume of 70 μl PBS. Navarixin (Cat# HY-10198; MedChemExpress) was given by oral gavage at 5 mg/kg 18 h after LPS administration and dissolved in 200 μl of vehicle (2.5% DMSO in corn oil).

### Intravital microscopy preparation for imaging the mediastinal visceral lung pleura

We anesthetized mice with ketamine/xylazine (60/20 mg/kg, i.p.), shaved their right chests, and performed tracheal intubation for mechanical ventilation with room air containing 1% isoflurane at 10 μl/g body weight delivered at 125 breaths per minute with 2.5 cm H_2_O positive end-expiratory pressure using a MiniVent system (Harvard Apparatus). Mice were then placed in the supine position, and an opening in the skin of the chest and underlying fascia was made to expose the right anterior ribcage. Ribs 2–4 were transected immediately to the right of the sternum and at posterior lateral locations and removed to make an opening in the ribcage, with point retractors placed to expose right lung lobes (Fig. 1 C). The inferior right lobe was repositioned with a saline-moistened cotton-tipped applicator so that its mediastinal pleural surface faced upward. The imaging window was then lowered over a pulmonary vein, and the application of negative pressure (−20 mmHg) was used to immobilize a segment of the lung against the coverslip.

### Microscopy

For intravital microscopy and for imaging lung sections, we used a Nikon A1r microscope with a CFI75 Apochromat 25XC water immersion objective and high-frequency HD25 resonance scanner (UCSF Biological Imaging Development CoLab). Fluorescent excitation was achieved using a Mai Tai DeepSea IR laser (950 nm) for multiphoton imaging and, where required, Coherent OBIS lasers (405, 488, 561, and 647 nm), with emitted light filtered through 440/80-, 525/50-, 600/50-, and 685/70-nm emission filters. The setup of imaging three-color channels achieved 2D image acquisition at 30.3 frames per second of 517 × 517 µm fields at 512 × 512 pixel resolution, or 7.7 frames per second of 344 × 344 µm fields at 1,024 × 1,024 pixel resolution. For 3D imaging studies, we used a Nikon AZ100 light-sheet microscope with an AZ-Plan Apo 2× NA 0.2 objective and Vortran Laser Launch providing excitation at 561 and 640 nm (UCSF Center for Advanced Light Microscopy, Fig. 1), a Nikon AXR with NSPARC confocal system with a CFI Plan Apochromat Lambda D 4× objective (Nikon, Fig. S1), and a Miltenyi Biotec UltraMicroscope Blaze light-sheet microscope system with a 1.1× MI Plan objective (Gladstone Institutes Light Microscopy Core, Fig. S4).

### Image analysis

ImageJ (National Institutes of Health) was used to measure lymphatic vessel diameters and to track cells, using manual cell tracking (Fig. 2 G) or Euclidean distance measurements (other analyses). Imaris (Oxford Instruments) was used to render 3D images and videos and to quantify valve opening rates and flow of cells in the lymph (total number of cells passing a prespecified point in the imaged lymphatic segment over a 10-min sampling period, quantified blinded to the treatment group).

### Immunofluorescence

For 3D imaging of fixed lungs (4% formaldehyde by tracheal inflation and immersion overnight), we used CUBIC clearing with immunostaining for GFP (Alexa Fluor 647–conjugated rabbit polyclonal, Cat# A-31852; Invitrogen), α-smooth muscle actin (αSMA, Cy3-conjugated clone 1A4, Cat# C6198; Sigma-Aldrich, or FITC-conjugated clone 1A4, Cat# F3777; Sigma-Aldrich), and, where relevant, mCherry (Alexa Fluor 594–conjugated clone 16D7, Cat# 74-000-3TP594; Invitrogen) for imaging entire lung lobes, as previously described (Takahashi et al., 2022). For imaging lung sections, 200-µm cryosections were prepared, stained, and imaged as described in our previous work (Cleary et al., 2020, 2024). Primary antibodies used were as follows: FITC-conjugated mouse anti-αSMA (clone 1A4, Cat# F37777; Sigma-Aldrich); rat anti-MHC-II (clone M5/114.15.2, Cat# 16-5321-81; Invitrogen), and goat anti-Ccl21 (Cat# AF457; R&D Systems) with the latter two unconjugated antibodies detected using cross-adsorbed donkey polyclonal secondaries: Alexa Fluor 647–conjugated anti-rat IgG and Cy3-conjugated anti-goat IgG (Cat# 712-605-153 and Cat# 705-165-147; Jackson ImmunoResearch, respectively).

### Metastatic melanoma model

As in previous reports (Headley et al., 2016; Ruhland et al., 2020; Ya et al., 2015), we gave *Rosa26*^mTmG^ mice an i.v. injection containing 1 × 10^5^ B16.F10-ZsGreen/mCherry cells for seeding pulmonary melanoma metastases.

### Preparations for imaging other organs

Mice were anesthetized as described above. For spleen and liver imaging, organs were flipped in a cranial direction to expose hilar structures, with the window placed on the border of organs and interstitial tissue. For imaging the heart, similar to previous approaches but without the use of glue (Lee et al., 2012), the heart tissue was exposed with a left-side thoracotomy and placing of the stabilization window over the left ventricle.

### Bronchoalveolar lavage (BAL) fluid analysis

The BAL fluid was collected by washing 1 ml of PBS in and out of the lungs three times. Cells were then pelleted by centrifugation (400 *g* for 4 min) and resuspended in PBS, 0.3% BSA, 0.5 mM EDTA, and 1 µg/ml Fc block (anti-CD16/32 clone 2.4G2, Cat# BP0307; Bio X Cell), stained with antibodies (anti-CD45-BV711, Cat# 103147; BioLegend; anti-SiglecF-BV605, Cat# 740388; BD; anti-CD11c-BV421, Cat# 117329; BioLegend) plus LIVE/DEAD Fixable Far Red Dead Cell Stain (Cat# L10120; Invitrogen), and assayed using a BD LSR II flow cytometer and FlowJo software. The protein content of the cell-free BAL supernatant was determined using a Pierce BCA protein assay (Cat# 23225; Thermo Fisher Scientific).

### Online supplemental material

Fig. S1 shows the anatomy of pulmonary vein–associated collecting lymphatics. Fig. S2 shows measurements of pulmonary lymphatic distension in LPS-induced acute lung inflammation. Fig. S3 shows imaging of pulmonary lymphatics in LPS-treated *PF4*-Cre:*Rosa26*^mTmG^ mice and *Prox1*-eGFP mice given PKH26 phagocytic cell linker (PKH26-PCL) to label alveolar macrophages. Fig. S4 shows 3D imaging of pulmonary lymphatics in metastatic melanoma. Fig. S5 shows stabilized imaging of lymphatic vessels draining the liver, spleen, and heart. Video 1 shows ventilation dependence of pulmonary lymphatic valve function and lung lymph flow. Video 2 shows the effect of changing ventilator tidal volume on lymph flow within pulmonary lymphatics following LPS-induced acute lung inflammation. Video 3 shows leukocyte rolling and adhesion within pulmonary lymphatics. Video 4 shows dendritic cells, monocytes/macrophages, and neutrophils in pulmonary lymphatics after LPS-induced acute lung inflammation. Video 5 shows the absence of alveolar macrophages and PF4+ cells from pulmonary lymphatics after LPS-induced acute lung inflammation. Video 6 shows the effect of pertussis toxin on leukocyte flow within pulmonary lymphatics following LPS-induced acute lung inflammation. Video 7 shows the effect of knockout of *Ccr7* on leukocyte flow within pulmonary lymphatics following LPS-induced acute lung inflammation. Video 8 shows the effect of *Ccr7* blocking antibody treatment on leukocyte flow within pulmonary lymphatics following LPS-induced acute lung inflammation. Video 9 shows the effect of navarixin treatment on leukocyte flow within pulmonary lymphatics following LPS-induced acute lung inflammation. Video 10 shows pulmonary lymphatic trafficking of leukocytes, cancer cell material, and cancer cells following lung metastasis of B16.F10 melanoma cells. Video 11 shows intravital imaging of lymphatics draining the liver, spleen, and heart. Data S1 contains a 3D model of the thoracic window used in these studies.

## Supplementary Material

Data S1 contains a 3D model of the thoracic window used in these studies.

## Data Availability

The 3D model needed to print the stabilization window used in this study is available as Data S1. Data supporting findings in this study are available from the corresponding author on request.
